# Stress Experience of COVID-19 Patients as Reported by Psychological Supporters in South Korea: A Qualitative Study

**DOI:** 10.3389/fpsyt.2022.834965

**Published:** 2022-03-29

**Authors:** Hyein Park, Nabin Lee, Jung Hyun Lee, Dayoung Lee, Kyoung Ae Kim, Hyun-Seung Kim, Eunhye Oh, Ji Hyun Ha, So Yoen Hyun, Juyeon Lee, Jiae Kim, Kyoungsun Jeon, Hyeong Taek Kim, Minyoung Sim

**Affiliations:** ^1^National Center for Disaster Trauma, National Center for Mental Health, Seoul, South Korea; ^2^Korea Trauma Research & Education Institute, Seoul, South Korea; ^3^Department of Psychiatry, Yongin Severance Hospital, Yongin, South Korea

**Keywords:** COVID-19, quarantine, stigma, psychological support, stress

## Abstract

**Background:**

COVID-19 patients experience various stressors during the quarantine period and after release from quarantine. However, stressors experienced during each period remain unclear.

**Methods:**

A total of 15 mental health experts from the integrated psychological support group for COVID-19participated in this study. Psychological support was provided for the total 932 confirmed COVID-19 patients and their families. Qualitative data were collected using Focus Group Interview (FGI). The participants were divided into two groups and semi-structured questions were used to allow participants to speak their minds.

**Results:**

During the quarantine period, difficulties of being diagnosed with COVID-19, concerns about recovery from COVID-19, stress related to quarantine, issues related to the treatment environment, and limited information about COVID-19 and communication were frequently reported. After release from quarantine, the reported main stressors include reinfection or reactivation, concerns about complications, and financial difficulties. Confusion as vectors and victims, stigma and discrimination, and conflicts within a family were observed during both periods.

**Conclusions:**

COVID-19 patients suffered various stressors during the quarantine period and after release from quarantine. Moreover, returning to their daily life required timely psychosocial support, intervention, and treatment for COVID-19 infection.

## Background

COVID-19 has become the worst pandemic in this century since the WHO reported its first case in December 2019 in China. The pandemic has continued for more than a year, steadily increasing the number of infected persons. During this period, there has been a considerable amount of interest in the mental health of COVID-19 patients (1, 2). Contracting COVID-19 could be traumatic in terms of threatened death or serious physical injury and accompany with shame and guilt, which can lead to social withdrawal, negative intrusive thought, post-traumatic stress disorder, and depression (3, 4).

Patients with infectious diseases suffer from various stressors such as longer quarantine duration, fear, boredom, inadequate supplies and information, financial loss, and stigma about the infection (5). Preventive measures, including social distancing, cross-border movement restrictions, lockdown, and self-quarantine, has impacted mental health globally. Moreover, emerging infectious diseases such as COVID-19 induce a lack of factual information, uncertainty about the epidemic trend, and continuity of the chain of events (6).

Furthermore, based on observation from previous outbreaks of Severe Acute Respiratory Syndrome (SARS) and the Middle East Respiratory Syndrome (MERS), providing psychological support for the confirmed patients was heavily emphasized during the pandemic. In addition, for effective intervention, it is necessary to identify the psychological problems of patients over the course of the disease (7). However, previous studies on stressors experienced by COVID-19 patients mainly focused on the contagious period (8–10).

National Center for Disaster Trauma (NCT) provided psychological support services for COVID-19 patients, quarantined individuals, and their families in South Korea. In this study, we aimed to find out stressors experienced by COVID-19 patients by analyzing the interviews of mental health experts who provided counseling to them. These experts provided psychological support and observed the patients during the quarantine period right after diagnosing COVID-19 and after release from quarantine. We hypothesized that the types of stressors would differ depending on under quarantine or after release from quarantine. Specifically, health and quarantine related issues would be prominent during the quarantine and secondary stressors such as financial difficulties would intensify after release from quarantine.

## Methods

### Participants

The integrated psychological support group for COVID-19 was established under the Ministry of Health and Welfare in January 2020. They provided mental health services, including 24-h hotline service and tele-counseling by mental health experts. They sent text messages containing information on mental health services and a self-rated screening tool to a list of COVID-19 patients given by the government. Psychological First Aid was provided through tele-counseling to those who called back to hotline service and for the high-risk group identified from mental health screening. For those who needed continuous counseling, psychological support was given from the quarantine period after diagnosing COVID-19 till after release from quarantine. A total of 15 mental health experts, including two psychiatrists, five psychologists, and seven social workers, provided tele-counseling to 932 COVID-19 patients and their families. All participants gave informed consent. They participated in psychological support for more than 3 months at the time of the interview.

### Data Collection

Qualitative data were collected using Focus Group Interview (FGI). The FGI is a method of interviewing a group of individuals at the same time, in which questions are freely discussed together and structured for research purposes. In the FGI, a moderator can also ask questions depending upon the situation (11).

Generally, to provide sufficient opportunity for participants to report, the size of FGI group is maintained as a minimum of 4 and a maximum of 12 individuals per group (12). We divided 15 participants into two groups and two mental health specialists oversaw each group and semi-structured questions were used to allow participants to speak their minds. The FGI was conducted once for each group and lasted 1 h. The questions included 10 open-ended ones (Table 1).

**Table 1:** Focus Group Interview (FGI) questions.

**Category**	**Question**
COVID-19 patients general	Was the COVID-19 patient cooperative with the counseling?
	Was the COVID-19 patient under quarantine at the time of counseling or after being completely cured?
Stressors of COVID-19 patients	What were stressors or difficulties reported by COVID-19 patients?
	When did COVID-19 patients report those stressors?; During or after quarantine
	Was the stressors reported during quarantine relieved after release from quarantine?
	Was there a change of stressors over time?
Psychological support	What helped with COVID-19 patients in terms of psychological support?
	Was there a change in the need for psychological support of COVID-19 patients over time?
	Did you experience the difficulties during psychological support activities?
	How did you cope with above difficulties?

The moderators played an active role in initiating interactions and discussions within the group. They familiarized themselves with the procedure and planned what to ask before the interview.

### Analysis Method

The analysis was carried out by two psychiatrists and one psychologist who had experience in conducting qualitative research. They had more than 2 years of work experience in disaster mental health and participated in providing psychological support for COVID-19 patients. The qualitative data were analyzed using content analysis. The content analysis classifies, abbreviates, and forms meaningful description of data collected to identify the phenomena of researchers' interest (13). The content analysis uses systematic procedures to increase objectivity, and to flexibly use deductive methods for constructing coding frames based on prior knowledge or theory and inductive methods for deriving categories and concepts from data (14).

Accordingly, we constructed a coding framework based on the existing literature on the mental health of COVID-19 patients and reconstructed the existing coding frameworks by categorizing relevant concepts from qualitative data. After pilot coding, triangular verification of coding was done and the process of recoding the review results and triangular verification was repeated. Triangular verification was conducted with the unanimous agreement of all the analysts and when anyone of the three analysts exhibited disagreement, we discussed to reach a consensus.

## Result

The results are summarized in Table 2.

**Table 2:** COVID-19 related stress divided by period.

**Period**	**Common stressors**	
During the quarantine	Being diagnosed with COVID-19	Confusion as vectors and victims Stigma and discrimination Conflicts within the family
	Concerns about recovery from COVID-19	
	Stress related to quarantine	
	Treatment environment issues	
	Limited information and communication	
After release from the quarantine	Reinfection or reactivation	
	Concerns about complications	
	Financial difficulties	

### The Quarantine Period due to COVID-19 Infection

#### Being Diagnosed With COVID-19

The confirmation of COVID-19 could be experienced as psychological trauma. Some patients had difficulty accepting the confirmation and expressed mistrust of testing results. They were pessimistic, pondering why they were infected out of all people, and showed anger toward the unidentified person who infected them.

“The most common responses were, ‘I am so unlucky', ‘Why does this happen to me?' etc.” (Participant 3).

#### Concerns About Recovery From COVID-19

Depression and anxiety escalated when quarantine release was delayed for 2–3 weeks. The patients were eager for recovery, sometimes despairing and even fearing death. In wards, the recovery level and release order were compared among patients. This made the patients more impatient.

“Patients considered hospitalization for 2 weeks as common, but after 3–4 weeks, they started to get worried about things like, ‘Is there something really wrong with me?' ‘Is it just me?' ‘Everyone else is getting discharged and will I be the only one who will not?' Patients who have been hospitalized for more than 3 weeks were worried about things like ‘What if I will not recover …' etc.” (Participant 5).

#### Stress Related to Quarantine

Patients suffered from loneliness, helplessness, and frustration during the quarantine period. They expressed utter helplessness since they were unable to do anything independently in the isolation ward and it was difficult to spend time meaningfully. Patients with pre-existing psychiatric illness showed fear of not receiving timely assistance due to quarantine and the exacerbation of symptoms. Prolonged quarantine often led to severe suffering.

According to the quarantine policy at the time of the interview, even asymptomatic COVID-19 patients were required to test negative for two consecutive PCR test to be released from quarantine. Therefore, the patients often continued quarantine for more than 20 days.

“The patient had been already in self-quarantine for 1 month due to close contact with another confirmed patient, and additional quarantine continued for 2 months after his confirmation… the patient said that ‘I'm so tired, I want to die, I want to kill myself, Should I jump out the window, I think I can only get out of here if I die”' (Participant 11).

#### Treatment Environment Issues

The change in the daily living environment was a stressor for COVID-19 patients. Staying in a decrepit facility for a long time and frequent transferring to different quarantine facilities intensified the stress even more. Further, one of the most common stressors in the treatment environment was the feeling of being watched, and the patients in hospital facilities experienced discomfort due to healthcare providers' frequent visits and observations.

“The facility is so old… it was suffocating enough…but there is no sunlight at all and the facility is so aged and I am being confined so it is so depressing… The nurse and healthcare providers keep coming and going and checking, so I could not sleep well and it was uncomfortable…” (Participant 5).

#### Limited Information and Communication

In the early days of the pandemic, the epidemiological characteristics of COVID-19 were unknown and response guidelines were not detailed. There was no accurate information about the process after the diagnosis. Increasing demand made it hard to contact the authorities for acquiring necessary information.

“The patient had to be transported after the confirmation but I think he was not given a detailed explanation. They did not say that they would come in the protective gear, so the patient said that he was so flustered when they showed up like that. They came in too suddenly and he was not prepared and conscious of how others would think about that, but they just came in and took him, so he was really flustered…” (Participant 5).

### After Release From the Quarantine

#### Reinfection or Reactivation

Concerns about COVID-19 infection continued even after complete recovery. Many patients regarded getting infected with COVID-19 as their vulnerability, which led to concern about reinfection of COVID-19. Some patients were worried that the virus would remain in their bodies for a long time and this anxiety was intensified as they were exposed to the press release on reinfection cases.

“As soon as the patient returned (to work), he started to show severe agitation. His hands were shaking and his heart was pounding on the day before going to work and in the morning, his hands were all sweaty and he was out of breath. He felt suffocated even at work so he had to get some air frequently. Similar symptoms appeared when using public transportation. I think his anxiety was closely related to worry about reinfection” (Participant 7).

Anxiety about reinfection or reactivation thwarted their daily lives. Some patients purchased all types of thermometers on the market and checked their body temperature often. Other patients avoided using public transportation or meeting people. They were concerned that they might spread the infection to other people, and because of this, they refrained from going outside or decided to go out only when neighbors were not around.

“Even after being cured, the patient repeatedly checked body temperature and was highly sensitive to the normal range. If it was any close to that threshold, anxiety level soared further …” (Participant 15).

#### Concerns About Complications

Various sequelae related to COVID-19 infection were reported, which ranged from physical symptoms such as fatigue and shortness of breath to vague somatic symptoms. Many patients became highly sensitive to all physical symptoms after COVID-19 infection.

“They were nervous that their health might suddenly take a downturn in an unexpected way and they might die suddenly. Some patients urged their family members to go to the emergency room in the middle of the night. They were easily overwhelmed by even minor physical symptoms. ‘Is there something wrong with my body?' ‘My lung seems to be damaged.' Hypochondriacal concerns have been commonly reported in many recovered patients” (Participant 11).

#### Financial Difficulties

Many patients were stressed out by financial difficulties as quarantine was prolonged. Patients who were daily employees, under temporary positions, and the sole breadwinners of their families, the quarantine caused severe financial difficulties.

“The patient returned to work for a week after the quarantine release, but his family tested positive again, so he had to undergo quarantine again. He lost all his business contacts and clients and did not know how to continue running the business…” (Participant 6).

### Over the Entire Period

#### Confusion as Vectors and Victims

Guilt feeling was commonly observed among the patients and they thought that they caused trouble and might have spread the infection to others. They felt sorry for those who underwent disinfection and self-quarantine because of them. However, they felt anger for being overly criticized since they were also victims who caught the virus unwittingly.

“The patient talked about a neighbor with a child living across her unit. She was worried that she might spread the infection to the kid, so she would listen to the sounds coming from outside and only go out cautiously when it is quiet…” (Participant 3).

“Patients were often hurt by the online malicious comments and were directly criticized by colleagues or close ones… On the one hand, they felt sorry for causing trouble to others, but on the other hand, they were resentful to those who were criticizing them without consideration of the unintended and unavoidable situation… They felt as if their whole life was degraded and considered relationships as meaningless…” (Participant 11).

#### Stigma and Discrimination

Patients were concerned that people would avoid or reject them if they disclose COVID-19 confirmation. For example, one patient who visited a hospital for non-COVID-19 symptoms was refused treatment due to the previous history of COVID-19 confirmation. Some patients experienced avoidance from their acquaintances and neighbors.

“After the treatment was over, I went to a community treatment center to submit an application for support payment, and someone said something like, ‘Hey, there comes a COVID-19 patient,' and I felt like being treated like a plague. Since then, I could not go to a community treatment center…” (Participant 14).

Discrimination and rejection were experienced even within close relations. The negative social attitude toward confirmed patients gave them a sense of self as a virus, bacterium, corpse, etc. They felt as if they were a toxic being to be avoided and such self-stigmatization harmed their self-esteem and self-efficacy.

“After being discharged, the patient wanted to visit an acquaintance, but the acquaintance kind of sounded like he was unwelcomed… So, he once again felt like he is treated like a bacterium by other people” (Participant 2).

Moreover, forced disclosure of personal information such as their paths and companions served as an excuse for criticism. In the case of mass infection involving religious facilities, gay bars, and mental hospitals, consequent stigma and discrimination were severely experienced.

“When public attention was focused on a particular group through media or online, patients were extremely anxious about being identified, and that was also evident in the counseling. They were afraid that the counselor would have a negative prejudice against them. It took them time to reveal their personal information and situation honestly…” (Participant 15).

#### Conflicts Within the Family

Furthermore, conflicts within the family increased due to infection and transmission, causing substantial damage and disruption to their daily life. The patients had mixed feelings toward their family, they felt sorry and were worried about their family and, at the same time, they felt lonely because the family members did not understand their difficulties.

“The patient quickly recovered from COVID-19 infection and seemed to be okay. Family members could not understand that patient would have some psychological difficulties. They would react like, ‘Why is it hard to return to work?' You only had minor symptoms but why do you keep complaining that you are having a hard time and need counseling?” (Participant 3).

“Some families were too sensitive to the patient. The family dissuaded him from returning to work to rest a little longer, and that just sounded annoying. They fought often. They realized that COVID-19 infection had taken a heavy toll on the whole family…” (Participant 7).

## Discussion

This study explored the psychological distress experienced by COVID-19 patients. As we hypothesized, during the quarantine, traumatic stressor, concerns about recovery, and the quarantine related difficulties were noticeable. Stressful treatment environment and limited information about COVID-19 and communication were also observed during this period. After release from quarantine, secondary stressors such as financial difficulties were remarkable as expected. It was noteworthy that concerns about the physical condition still continued after the release from quarantine. Consistent with previous studies of infectious diseases, ‘vector or victim' issue and suffering from stigma were reported over entire period (15).

### Fear About Health Deterioration

Patients with COVID-19 reported concerns about the exacerbation of the disease, recurrence, unpredictable complications, and even death (9). The daily lives of confirmed patients were greatly affected by preoccupied concerns and anxiety to the extent that the term “COVID-19 health anxiety” is coined (16). They repeatedly checked their body temperature and were reluctant to use public transportation or meet people. This is consistent with previous findings that patients with infectious diseases show health behavior changes such as excessive hand washing and avoiding closed places even after recovery (17, 18).

Moreover, COVID-19 patients suffer from long-lasting symptoms, such as fatigue, headache, loss of smell, and shortness of breath even after recovery (19). Some patients became sensitive to small body symptoms because of their worry about complications (20).

### Stress Related to Quarantine

Quarantine or isolation causes psychological difficulties such as loneliness and helplessness (5, 21). Several studies have shown that quarantined persons are more likely to develop depression, irritability, insomnia, post-traumatic stress symptoms, and emotional exhaustion than those who were not quarantined (5, 22).

The psychological disturbance became prominent as the quarantine period was prolonged (9, 10, 23). According to prior study of COVID-19 patients admitted to a community treatment center (CTC) in Korea, only 4.3% of COVID-19 patients had depression at the beginning of quarantine, which increased to 15.6% after 4 weeks (24). A longer quarantine period was related to more emotional and psychological distress (5, 24). It was also a risk factor for post-traumatic stress disorder (25).

In South Korea, quarantine release criteria were changed from test-based to symptom-based, and the average quarantine period was shortened by 10 days. Accordingly, the reports of quarantine stress among patients decreased. The reduction of the unnecessary quarantine period is necessary for maintaining good mental health (5).

### Vector or Victim

Being treated as a vector and a victim is a unique feature of infectious diseases (15). A considerable number of COVID-19 patients expressed guilt that they might have spread the infection to their families or others and at the same time, they showed resentment at being criticized without being considered as victims (15, 26). Another qualitative study has also shown that COVID-19 patients suffered from guilt that they were infected and infected others due to their carelessness (26). Given that it is difficult to identify the source of the infection and that asymptomatic infection of COVID-19 is frequent, attributing it to one's own responsibility would be improper in many cases.

Patients feel ashamed of themselves as if they were defective, which is exacerbated by the stigma of COVID-19 (27). Some patients faced disadvantages at work. They were criticized by the people around them. Moreover, they faced difficulty returning to work and society even after recovery, leading them to financial difficulties. Previous studies showed that financial problems, stigma, and discrimination caused stress even after release from quarantine (22).

As well as the impact of maladaptive guilt and shame on mental health (3, 27), the stigma of infected persons caused barriers in testing and diagnosing, which lead to the spread of COVID-19 (28). It also interrupted proper follow-up treatment.

Stress caused by stigmatization cannot be improved by psychological counseling, and it requires accurate government policies to prevent them. A national community-based anti-stigma and advocacy activity could significantly decrease mental health and public health problems, including violence, self-harm, and suicide (29). Reducing the social stigma of patients will help them to return to their daily life without any psychological problems and adjust to their daily life.

### Information Delivery

Recognizing and responding to infodemic was one of the most important strategies used for managing COVID-19 pandemic (30).The fear of an unknown illness leads to increased anxiety and sharing of misinformation with unknown sources (31). Therefore, providing information about diagnosis and treatment procedures, psychological education including stress management, and hotline services to the public could have been helpful.

Furthermore, less knowledge of diseases has a strong link to discrimination and stigma. Hence, efforts should be taken to protect the public from fake news and provide accurate information to control stigma and fear of infection (32–35).

Moreover, factual and transparent information should be provided through official narratives, online news, social media, and local government to the public (36). In addition, since information can be interpreted differently depending on the political orientation, it is necessary to provide accurate information separately from political communication (37).

### Practical Implication

#### Advice for Mental Health Professionals

Immediately after confirmation of an infectious disease, it is easy to be mentally overwhelmed because several stressors occur at once, such as fear of death, deterioration of health, infection with others, and difficulties caused by quarantine, etc. They are often confused whether they are vectors or victim, and it might be hard to report psychological difficulties because they are guilty and ashamed. Therefore, clinicians should be able to fully understand the difficulties that patients with infectious disease face at the beginning of confirmation and actively provide psychological support (38, 39). In addition, normalization that anxiety of re-infection or complication may continue for a while even after quarantine could promote their psychological recovery.

#### Advice for Policy Maker

WHO emphasized the management of mental health among essential health services to be guaranteed during COVID-19 public health emergencies (40).

As can be seen from the results of this study, COVID-19 patients suffer a lot even after release from quarantine and return to daily life. The long-term effects of infectious diseases has been found as high levels of depression, anxiety, and post-traumatic stress disorder a year after SARS pandemic (41). Disaster-related suicides are said to be on the rise over the next 2–3 years after the disaster (42, 43). In addition to taking mid- to long-term mental health recovery plans, funding for mental health is required (44).

### Limitations and Suggestions

This study has several limitations that need to be addressed. In this study, the stress experience of patients was examined through the report of mental health professionals who provided psychological support to them. This is delivered in the language of an experts who provided psychological support rather than directly translating the words of patients. Hence, there might be a bias in the classification system because the stress experience of patients might differ from the practitioner's point of view. However, the problem was reported objectively and accurately since practitioners had prior knowledge of disaster stress experience. Furthermore, psychosocial support was given through telephone counseling, instead of face-to-face counseling. Previous study reported that there is no significant difference in effectiveness between face-to-face and telephone counseling (45), but non-verbal communication restrictions can make it difficult to track the problems of patients in depth. Moreover, in this study, the stress level of patients was not periodically traced in a detailed manner. A longitudinal study of patients' experiences in the future may help us understand the long-lasting stressors of patients with infectious diseases. In addition, our data were collected and analyzed in the early stages of COVID-19. Considering that the quarantine guidelines were frequently changed and the quarantine period was longer, the level of anxiety and stress might have increased at a later stage. In addition, the issue of personal information disclosure and stigma of patients was severe. The pattern of early outbreaks and current trends differ in many aspects. Hence, stress experiences must be analyzed periodically for identifying and responding to the long-term effects of the epidemic.

## Conclusion

COVID-19 patients experienced various stressors from the moment they were confirmed. The stressors continued even after recovery. Patients had a confusing experience of being treated as both vectors and victims after being confirmed with COVID-19.Stigma and discrimination were important issues over the entire period. During the quarantine period, thoughts about the infection and isolation mainly caused stress. After their release from quarantine, the patients were troubled with concerns about sequelae and reinfection, and financial difficulties.

## Data Availability Statement

The original contributions presented in the study are included in the article/supplementary material, further inquiries can be directed to the corresponding author/s.

## Ethics Statement

The studies involving human participants were reviewed and approved by Institutional Review Board of National Center for Mental Health. The patients/participants provided their written informed consent to participate in this study.

## Author Contributions

NL, JHL, MS, and DL conceived and designed the study. NL, HK, SH, KK, H-SK, EO, JH, and JL collected the data. NL, JHL, and HP analyzed and interpreted the data. HP, JHL, and MS drafted and wrote the manuscript. MS, JHL, DL, JK, and KJ contributed to critical revision of the article. All authors read and approved the final manuscript.

## Conflict of Interest

The authors declare that the research was conducted in the absence of any commercial or financial relationships that could be construed as a potential conflict of interest.

## Publisher's Note

All claims expressed in this article are solely those of the authors and do not necessarily represent those of their affiliated organizations, or those of the publisher, the editors and the reviewers. Any product that may be evaluated in this article, or claim that may be made by its manufacturer, is not guaranteed or endorsed by the publisher.
